# IFN-gamma and TNF associated with severe falciparum malaria infection in Saudi pregnant women

**DOI:** 10.1186/1475-2875-13-314

**Published:** 2014-08-13

**Authors:** Amre Nasr, Gamal Allam, Osama Hamid, Abdelhamid Al-Ghamdi

**Affiliations:** Department of Microbiology, College of Medicine, Taif University, PO Box 888, Taif, Saudi Arabia; Department of Basic Medical Sciences, College of Medicine, King Saud bin Abdulaziz University for Health Sciences, Riyadh, Saudi Arabia; Department of Microbiology, Faculty of Science and Technology, Al-Neelain University, Khartoum, Sudan; Department of Zoology (Immunology Section), Faculty of Science, Beni-Suef University, Beni-Suef, Egypt; Department of Public Health, Jazan Health Affairs- District MoH, Jazan, Saudi Arabia; Department of Surgery, College of Medicine, Taif University, Taif, Saudi Arabia

**Keywords:** Severe malaria, TNF, IFN-γ, Cytokines, Gene polymorphism

## Abstract

**Background:**

Tumour necrosis factor (TNF) and interferon gamma (IFN-γ), encoded by *TNF*-836 C/A (rs 1800630) and *IFN*-*γ* -1616 C/T (rs2069705) genes, are key immunological mediators that are believed to both play protective and pathological roles in malaria. The aim of this study was to investigate the relationship between *TNF*-836 C/A and *IFN*-*γ*-1616 C/T polymorphism and susceptibility to severe malaria in pregnant women.

**Methods:**

A prospective cohort (cross-sectional) study was conducted in pregnant women attending the out-patient clinic in King Fahad Specialist Hospital in Jazan (KFSHJ), with a clinical diagnosis of malaria. A total of one hundred and eighty six pregnant women were genotyped for single nucleotide polymorphism (SNP) for *TNF* and *IFN*-*γ* using Taqman® MGB Probes. Serum cytokine concentrations were measured by sandwich ELISA method.

**Results:**

A hospital case–control study of severe malaria in a Saudi population identified strong associations with individual single-nucleotide polymorphisms in the *TNF* and *IFN*-*γ* genes, and defined *TNF*-836 C and *IFN*-*γ*-1616 T genotypes and alleles which were statistically significantly associated with severe malaria infection. Furthermore, *TNF*-836 CC and *IFN*-*γ*-1616 TT genotypes were associated with higher serum concentration of TNF and IFN-γ, respectively, and with susceptibility to severe malaria.

**Conclusions:**

This data provides a starting point for functional and genetic analysis of the TNF and IFN-γ genomic region in malaria infection affecting Saudi populations.

## Background

Malaria is one of the most important and prevalent infectious diseases in the world. The World Health Organization (WHO) estimated 219 million malaria cases worldwide with 660,000 deaths due to *Plasmodium* infection per year [1]. Women in endemic areas become highly susceptible to malaria during their first and second pregnancies, despite immunity acquired after years of exposure [2]. These effects are most highly associated with *Plasmodium falciparum* infections of the placenta referred to as placental malaria (PM) [3]. Malaria infection during pregnancy also increases the risk of moderate/severe maternal anaemia, which is a recognized risk factor leading to low birth-weight (LBW) [4]. As there are over 85 million pregnant women at risk of *P. falciparum* infection every year [5], there is a need to better understand the mediators of poor clinical outcome of pregnancy-associated malaria (PAM) [6]. Both *P. falciparum* and *P. vivax* infections can cause adverse pregnancy outcomes, including maternal anaemia and LBW due to preterm delivery and foetal growth restriction, but the underlying mechanisms could differ [7].

Host susceptibility to malaria is attributable to a number of factors, which include the genetic background of both host and pathogen. Indeed host genetic factors are involved in the regulation of the individual’s immunological competence. Several chromosomal regions containing genes coding for cytokines or cytokine receptors have been implicated in the control of *P. falciparum* infection levels [8]– [10].

Substantial increase in tumour necrosis factor (TNF) [11]– [14], and interferon gamma (IFN-γ) [11, 15], have been found in placental blood or tissue in response to malaria infection. These cytokines are known to aid in the elimination of parasites from the placenta by enhancing phagocytic activity of macrophages, generating reactive oxygen intermediates and L-arginine-derived nitric oxide, and stimulating the proliferation of T cells. Thus, Th1-type responses are of parasitological importance. However, overproduction can jeopardize the pregnancy, as Th1 response is associated with maternal anaemia, spontaneous abortions, and premature deliveries [16].

Host genetic factors have been shown to influence malaria infection intensity and clinical malaria. Several candidate genes have been associated with resistance against severe malaria, whereas linkage or association analyses mapped several loci controlling mild malaria and/or parasitaemia [17].

The first aim of this study was to examine TNF and IFN-γ cytokine concentrations in pregnant women with a clinical diagnosis of malaria. Those women were attending the outpatient clinics at King Fahad Specialist Hospital in Jazan (KFSHJ), Saudi Arabia. The second aim of this study was to investigate whether *TNF* −836 C/A (rs 1800630) and *IFN*-*γ*-1616 C/T (rs2069705) genotypes and alleles frequency, are associated with susceptibility to either uncompleted or severe malaria infection among Saudi women living in the southern part of Saudi Arabia. The third aim of the study was to examine the association between single nucleotide polymorphism (SNP) in promoter regions of *TNF* and *IFN*-*γ* and cytokine levels.

## Methods

### Study area

This study was conducted in Jazan city in the southern Kingdom of Saudi Arabia (KSA), during the dry season, March 2012 to August 2013. The study area was previously described in detail by Nasr *et al*. [18].

### Study design and patient enrolment

A prospective cohort (cross-sectional) study was conducted in pregnant women attending the out-patient clinic in King Fahad Specialist Hospital in Jazan (KFSHJ), with a clinical diagnosis of malaria. Patients with symptomatic malaria are those with one or more of the classical symptoms of malaria infection as described by the WHO [19]. Patients were recruited after written informed consent was gained from them. Blood samples were taken for confirmation of malaria. Once patients were found to be positive for malaria, treatment was offered. Patients with symptomatic malaria (Uncomplicated Malaria, UM) in their first trimester of pregnancy received quinine (20 mg/kg loading dose) plus clindamycin for seven days. Patients in their second and third trimesters received ACT. Standard ACT was artemisinin (4 mg/kg/day) given on days 0–2 and a single dose of sulphadoxine-pyrimethamine (25 mg/kg) given on day 0 [20]– [22]. Patients with severe malaria (SM) received artesunate plus quinine (10 mg/kg/8 hours). All patients were reviewed by the study gynecologist to confirm that there were no other co-infections apart from malaria. No one from the study groups was found to be HIV positive. All patients with other infectious diseases were excluded from the study. The study is part of a longitudinal research and is described previously by Nasr *et al*. [18]. The study was reviewed and approved by the Ethics Review Committee in the College of Medicine at Taif University.

### Sample collection

Before pharmacological treatment was started, 3 ml of venous blood sample was collected into EDTA Vacutainer® tubes (Becton Dickinson, Meylan, France) for parasite measurements. This was put into thick and thin blood smears obtained at the time of collection, which were then stained with Giemsa. The blood samples were separated and plasma samples were stored at −80°C until used for the cytokines analysis.

### Microscopic observation

Thick and thin films were made for microscopic examination using standard Giemsa staining. Parasite densities were determined by counting the number of parasites per 1,000 erythrocytes in thin film.

### Serum and DNA extraction

Genomic DNA was purified from blood using a commercial DNA purification kit QIAmp® DNA blood mini kit (Qiagen, Hilden, Germany) [23].

### Parasite genotype

Five μl of DNA samples were used to detect the *P. falciparum* malaria parasite using a polymerase chain reaction (PCR), targeting for *msp2* (FC27) clone as described in details below. Nested PCR was performed to determine the numbers of *msp2* (FC27) clone. Amplifications were done in 10 μl reaction mixture containing DNA template, iProof™ High-Fidelity Master Mix (BIO-RAD Laboratories, Hercules, CA) and 500 nM of primer pairs. The methodology and sequences of used primers have been presented in detail elsewhere in [24] and [25], respectively. For the negative controls, blood samples were collected from Swedish individuals who were never exposed to *P. falciparum* malaria parasites. For the positive controls, blood samples were collected from Sudanese individuals who have been exposed to *P. falciparum* malaria parasites in the past.

### Cytokines genotyping

Single nucleotide polymorphism (SNP) for *TNF*-863 C/A (rs1800630) and *IFN*-*γ* -1616 C/T (rs2069705) were analysed with Taqman® MGB Probes from Applied Biosystems according to the manufacturers protocol as previously mentioned in [26, 27].

### Cytokine concentrations

Serum cytokine concentrations were measured by the sandwich ELISA method [11, 28]. The following Abs were used: IFN-γ, mAb 1-D1K and mAb 7-b6-1-biotin (MABTECH, Nacka, Sweden); TNF, mAb, and polyclonal Ab (Genzyme, Cambridge, MA). Detection limits for cytokines were: IFN-γ and TNF 1.0 pg/ml [11].

### Statistical analysis

The distribution of cytokine [TNF and IFN-γ] genotype, allele’s frequencies and [TNF and IFN-γ] concentration were analysed using SPSS version 16.0 (SPSS, Inc, Chicago, IL, USA). Logistic regression analyses were performed to assess associations of genotype (dependent variable) and risk of malaria severity. Associations were quantified using odds ratios [OR] with 95% confidence intervals (CI), which when they do not cross 1.00, it is defined as statistically significant. The [TNF and IFN-γ] heterozygote group were used as a reference in the analyses. Using the same software to perform an overall comparison of allele frequency using a 2 × 2 chi-square test. Differences in cytokine [TNF and IFN-γ] concentrations between different study groups were analysed using *Kruskal*-*Wallis* test and *P*- value will be corrected for ties. Logistic regression analyses were performed to assess the association between the cytokine [TNF and IFN-γ] serum levels in individuals with different cytokine genotypes.

## Results

### Study participant characteristics

A total of one hundred and eighty six pregnant women, were consecutively enrolled in the study and were classified into three groups according to malaria outcome. Group I: Malaria free control (MFC), were malaria parasite negative and had no clinical symptoms of malaria infection [n = 60 (32.3%); median age of 22 with a range of (18–26) years] (Table 1). Group II: Uncomplicated malaria patients (UM) were malaria parasites positive and had one or more of the classical symptoms of malaria infection [n = 62 (33.3%); median age of 22 with a range of (18–26) years]. The median of parasite density was 1,200 with a range of 250–2,000 parasite/μl. This is a relatively low parasite density according to the WHO criteria [19] (Table 1). Group III: Severe malaria patients (SM) [n = 64 (34.4%); median age of 20 with a range of (18–22) years] were malaria parasites positive and fulfilled one or more of the WHO criteria for severe (complicated) *P. falciparum* [median of parasite density was 2,400 with a range of 2,000-2,800 parasite/μl) (Table 1). The serum concentration of TNF and IFN-γ cytokines were statistically significantly higher in SM patients compared to other groups (Overall *P* value <0.001 for both cytokines) (Table 1). Overall, there was statistical difference in the median and range of mother's age in malaria outcome *P* value <0.001 (Table 1).

**Table 1:** **Description the median and range of parasitological**, **cytokine** (**TNF**, **IFN**-**γ**), **age and Hb among the study group**

Diagnosis	Trimester		Parasite	TNF	IFN-γ pg/ml	Age	Hb gm/dl
				pg/ml			
**MFC n** = **60**	First trimester n = 13	Median		1.76	2.83	21	14
		Range		1.44- 2.17	2.01- 2.85	18-26	10- 16
	Second trimester n = 13	Median		1.87	2.83	22	13
		Range		1.36- 2.08	2.73- 3.26	18-26	10- 16
	Third trimester n = 34	Median		1.81	2.84	23	14
		Range		1.38- 2.21	2.01- 2.98	18- 26	10- 16
*P* value^Ψ^ within the subgroups				0.370	0.283	0.129	0.451
**UM n** = **62**	First trimester n = 43	Median	1300	5.65	4.37	23	13
		Range	250- 2000	5.14- 6.10	2.64- 5.38	18-26	12-14
	Second trimester n = 12	Median	1150	5.60	4.72	22	13
		Range	510- 2000	5.30- 6.03	2.70- 5.37	18-26	12- 14
	Third trimester n = 7	Median	600	5.66	4.63	21	13
		Range	320- 650	5.58- 5.78	2.63- 5.36	18-26	12- 14
*P* value^Ψ^ within the subgroups			0.008	0.797	0.478	0.944	0.573
**SM n** = **64**	First trimester n = 46	Median	2400	7.95	7.51	20	10
		Range	2000- 2800	5.72- 43.64	5.40- 19.50	18-22	8- 13
	Second trimester n = 14	Median	2400	8.35	7.57	20	9.5
		Range	2000- 2800	6.22- 21.28	5.52- 16.22	18-22	8- 12
	Third trimester n = 4	Median	1250	7.16	6.65	20	10
		Range	1000- 2000	6.39- 8.66	5.60- 7.66	19- 22	8.9- 12
*P* value^Ψ^ within the subgroups			0.383	0.635	0.565	0.771	0.754
Overall *P* value* between the groups			<0.001	<0.001	<0.001	0.001	0.001

^**Ψ**^ *P* value was calculated between the groups using one way ANOVA test.

*P value was calculated between the groups using non-parametric test (*Kruskal*-*Wallis*).

Overall, there was statistical difference in the median of the parasite density which was significantly higher in SM compared to UM patients (*P* value <0.001, Table 1). All women attending the outpatient clinic at the time of enrolment were analysed initially by microscopy of blood film, and subsequent confirmation by PCR as mentioned in parasite genotype section.

The characteristics of the pregnant women are shown in Table 1. The prevalence of malaria parasites density during the pregnancy duration were statistically significantly higher in pregnant women during the first and second trimesters when compared with the pregnant women in their third trimester; overall *P* value = 0.008 (Table 1). However, no statistical difference was seen between the first and second trimester regarding the prevalence of parasites density, which were observed between SM (*P* value = 0.383; Table 1).

### Description of gravid, ABO blood group and malaria complications among the study groups

One of the findings is that younger women (20 years old) were at higher risk of severe malaria than older women; overall *P* value = 0.001 (Table 1). The prevalence of malaria infection in the pregnant women with primigravidae was higher than those with multigravidae (*P* value <0.001) (Table 2). Pregnant women carrying O blood group were associated with severe malaria compared with other ABO blood groups (overall *P* value <0001, Table 2).

**Table 2:** **Description of gravid**, **ABO blood group and malaria complications among the study groups**

Dependant variables	MFC	UM	SM	***P***value
	n = 60 (32.3%)	n = 62 (33.3%)	n = 64 (34.4%)	
**Gravidae**				<0.001
Primigravidae	4 (6.7%)	54 (87.1%)	56 (87.5%)	
n = 114 (61.3%)				
Multigravidae	56 (93.3%)	8 (12.9%)	8 (12.5%)	
n = 72 (38.7%)				
**ABO**				<0.001
O n = 124 (66.7%)	44 (73.3%)	37 (59.7%)	43 (67.2%)	
A n = 20 (10.8%)	8 (13.3%)	4 (6.5%)	8 (12.5%)	
B n = 30 (16.1%)	4 (6.7%)	18 (29%)	8 (12.5%)	
AB n = 12 (6.5%)	4 (6.7%)	3 (4.8%)	5 (7.8%)	
**Malaria complications**				<0.001
No complications	60 (100%)	62 (100%)	0 (0%)	
Anaemia	0 (0%)	0 (0%)	49 (76.6%)	
Cerebral malaria	0 (0%)	0 (0%)	8 (12.5%)	
Hypoglycaemia	0 (0%)	0 (0%)	7 (10.9%)	
**Treatment**				<0.001
No treatment	60 (100%)	0 (0%)	0 (0%)	
Quinine	0 (0%)	43 (69.4%)	0 (0%)	
ACT*	0 (0%)	19 (30.6%)	0 (0%)	
Artesunate plus quinine	0 (0%)	0 (0%)	64 (100%)	

*Artemisinin-based combination therapy (ACT).

Table 2 shows that 76.6% of SM patients had severe normocytic anaemia (haemoglobin <7 g/dl, and haematocrit <20%) due to the malaria infection which was statistically significantly higher compared to those with cerebral malaria (unrousable coma), convulsions (more than two per 24 hours), (12.5%) and hypoglycaemia (blood glucose <2.2 mmol/l) (10.9%); overall *P* value <0.001 (Table 2). Among 62 pregnant women diagnosed with UM, 43 (69.6%) in the first trimester were treated by quinine compared with 30.4% pregnant women in their second and third trimester of pregnancy who were treated with artemisinin-based combination therapy (ACT). All 64 patients with severe malaria were treated with artesunate plus quinine (overall *P* value <0.001, Table 2). There were no deaths within the cases of UM or SM malaria noted during the study.

### *TNF*-863 C/A and *IFN*-*γ*-1616 *C*/*T*; genotypes and alleles

The *TNF*-863 C/A and *IFN*-*γ*-1616 C/T genotype and allele frequencies amongst the control group and the patients of SM and UM were found to be in Hardy-Weinberg equilibrium (for *TNF*-863 C/A genotype and allele; *P* value = 0.61 and 0.33, respectively) and for (*IFN*-*γ*-1616 C/T genotype and allele; *P* value = 0.57 and 0.21, respectively) (Table 3). The overall *TNF*-863 C/A genotypic and allelic frequencies differed statistically significantly between SM and UM patients compared to the MFC group [Overall genotypic frequency; Odds Ratio [OR] = 6.75, 95% Confidence Interval [CI] = (1.35- 33.77), *P* value = 0.020] and [Overall allelic frequency; OR = 2.15, 95% CI = (1.54- 4.69), *P* value = 0.001] (Table 3). The overall *IFN*-*γ* -1616 C/T genotypic and allelic frequencies differed statistically significantly between SM and UM patients compared to the MFC group [Overall genotypic frequency; OR = 8.50, 95% CI = (2.19- 12.95), *P* value = 0.002] and [Overall allelic frequency; OR = 1.76, 95% CI = (0.12- 3.95), *P* value = 0.014] (Table 3).

**Table 3:** **Distribution and logistic regression analysis of the cytokine** (**TNF**, **IFN**-**γ**) **genotypes and allele frequency in relation to severe** (**SM**) **compared with uncomplicated malaria** (**UM**) **and malaria free control** (**MFC**)

Dependent variable	MFC	UM	SM	Hardy-Weinberg equilibrium test
	n = 60(%)	n = 62(%)	n = 64(%)	
***TNF*** −**863 C**/A*				
CC	6 (6.5%)	32 (34.8%)	54 (58.7%)	0.61
CA	27 (47.4%)	22 (38.6%)	8 (14.0%)	
AA	27 (73%)	8 (21.6%)	2 (5.4%)	
**Alleles frequency**‡				
C	0.32	0.69	0.91	0.33
A	0.62	0.31	0.09	
***IFN***-***γ*** -**1616 T**/**C** ^¥^				
TT	2 (2.6%)	24 (31.2%)	51 (66.2%)	0.57
CT	35 (49.3%)	26 (36.6%)	10 (14.1%)	
CC	23 (60.5%)	12 (31.6%)	3 (7.9%)	
**Alleles frequency** ^£^				
T	0.33	0.60	0.88	0.21
C	0.67	0.40	0.12	

*Overall genotypic frequency; Odds Ratio [OR] = 6.75; 95% Confidence Interval [CI] = (1.35- 33.77), *P* value = 0.020.

‡Overall allelic frequency; OR = 2.15; 95% CI = (1.54- 4.69), *P* value = 0.001.

^¥^Overall genotypic frequency; OR = 8.50; 95% CI = (2.19- 12.95), *P* value = 0.002.

^£^Overall allelic frequency; OR = 1.76; 95% CI = (0.12- 3.95), *P* value = 0.014.

Logistic regression analysis revealed that the *TNF* −863 CC genotype is significantly higher amongst SM group compared to the UM group [OR = 4.64, 95% CI = (1.85- 11.64), *P* value <0.001] (Table 4). The frequency of the C-allele was statistically significantly dominant in SM patients compared to the UM patients [69% for C-allele in UM *versus* 91% for C-allele in SM; OR 0.24, 95% CI (0.07- 0.68) and *P* value = 0.003] (Tables 3 and 4).

**Table 4:** **Logistic regression analysis of** ***TNF***-**863 C**/**A genotype and allele frequency among SM compared with UM patients**

***TNF***−863 C/A	OR^‡^(95% CI)	***P***value
Genotypes		
CC	4.64 (1.85-11.64)	<0.001
CA	1.00	
AA	0.69 (0.12- 3.94)	0.67
**Alleles frequency**		
C	0.24 (0.07- 0.68)	0.003
A		

^‡^OR represent odds ratios while CI represents confidence intervals. Uncomplicated malaria patients (UM) were assigned 0 while severe malaria (SM) patients were assigned 1 in the logistic regression analysis. OR above 1 represented value associated to SM patients while less than 1 value represented to UM patients.

Logistic regression analysis showed that the *IFN*-*γ*-1616 TT genotype is significantly higher amongst SM group compared to the UM group [OR = 5.53, 95% CI = (2.30- 13.27), *P* value <0.001] (Table 5). The frequency of the T-allele was statistically significantly dominant in SM patients compared to the UM patients [60% for T-allele in UM *versus* 88% for T-allele; OR 0.24, 95% CI (0.07- 0.68) and *P* value = 0.003] (Tables 3 and 4).

**Table 5:** **Logistic regression analysis of** ***IFN***-***γ*** -**1616 T**/**C genotype and allele frequency among SM compared with UM patients**

***IFN***-***γ***-1616 T/C Genotypes	OR^‡^(95% CI)	***P***value
TT	5.53 (2.30- 13.27)	<0.001
TC	1.00	
CC	0.65 (0.15- 2.80)	0.56
**Alleles frequency**		
T	0.28 (0.10- 0.76)	0.008
C		

^‡^OR represent odds ratios while CI represents confidence intervals. Uncomplicated malaria patients (UM) were assigned 0 while severe malaria (SM) patients were assigned 1 in the logistic regression analysis. OR above 1 represented value associated to SM patients while less than 1 value represented to UM patients.

### Association of *TNF*-863 C/A, *IFN*-*γ*-1616 C/T genes polymorphism and serum TNF, IFN-γ concentration

The relationship between the *TNF*-863 C/A polymorphism and serum TNF concentration was analysed regardless of the differences in the study groups. As shown in Table 6, the homozygous *TNF*-863 CC genotype is significantly associated with higher levels of TNF amongst the whole study groups compared to the heterozygous *TNF*-863 CA genotype [OR = 4.04, 95% CI = (2.01- 8.13), *P* value <0.001] (Table 6). In contrast, the homozygous *TNF*-*863* AA genotype is significantly associated with lower levels of TNF in the combined study population compared to the heterozygous *TNF*-863 CA genotype [OR = 0.31, 95% CI = (0.11- 0.86), *P* value = 0.024] (Table 6).

**Table 6:** **Logistic regression analysis of** ***TNF*** −**863 C**/**A and** ***IFN***-***γ*** -**1616 T**/**C genotypes in association to TNF and IFN**-**γ concentration** (**pg**/**mL**) **respectively in the combined study groups**

Genotypes	OR^‡^ (95% CI)	***P***value
***TNF*** −**863**		
CC	4.04 (2.01- 8.13)	<0.001
CA	1.00	
AA	0.31 (0.11- 0.86)	0.024
***IFN***-***γ*** -**1616**		
TT	8.49 (4.03- 17.91)	<0.001
TC	1.00	
CC	0.81 (0.33- 1.92)	0.002

^‡^OR represent odds ratios while CI represents confidence intervals. Lower levels of TNF (mean ± SD; 2.99 ± 1.24) and IFN-γ (4.01 ± 1.94) concentration were assigned 0 while severe malaria (SM) patients were assigned 1 in the logistic regression analysis. OR above 1 represented value associated to SM patients while less than 1 value represented to UM patients.

The association between the *IFN*-*γ* -1616 C/T polymorphism and the concentration of IFN-γ was analysed regardless of the difference in the study groups. The homozygous *IFN*-*γ* -1616 TT genotype is significantly associated with higher concentration of IFN-γ compared to the heterozygous *IFN*-*γ* -1616 TC genotype [OR = 8.49, 95% CI = (4.03- 17.90), *P* value <0.001] (Table 6). However, the homozygous *IFN*-*γ* -1616 CC had no influence in the concentration of serum IFN-γ amongst the study population compared with the heterozygous *IFN*-*γ* -1616 TC genotype [OR = 0.81, 95% CI = (0.33- 1.92), *P* value = 0.610] (Table 6).

## Discussion

The present study suggests that the prevalence malaria infection is higher in pregnant women during the first trimester compared to second and third trimesters. This finding was reported previously in the same study area [18]. The data presented here suggests that the youngest mothers, who are less than 21 years old, are more susceptible to severe malaria compared to other age groups. A previous study in Mali suggested that young age is an important risk factor for malaria infection in pregnant women [29]. Thus, control measures against malaria infection should target younger rural women in their first trimester of pregnancy [29].

The data presented here suggests that the prevalence of malaria parasite’s density during the pregnancy duration were statistically significantly higher in pregnant women during the first and second trimesters. A previous study suggested that the peaks of peripheral parasitaemia is around 8 to 15 weeks of gestation [30]. In the present study, the prevalence of malaria infection in the primigravidae was higher than those who were multigravidae. This corresponds well with the finding that IgG responses [variant surface antigens specific to pregnancy associated malaria (VSA_PAM-_specific)] appear around weeks 18 to 20 in primigravidae and somewhat earlier in multigravidae [31, 32].

The present study found that pregnant women carrying the O blood group were associated with severe malaria compared with those non-O blood group carriers. Several studies from Gabon, Gambia, Malawi and Sudan [33]– [36] suggested that there is a tendency towards a protective effect of the blood group O against placental malaria infection regardless of parity; however this was not statistically significant. The higher risk of placental parasitaemia in primiparae with blood group O that was found in two previous studies could not be confirmed. The significant association of blood group O with placental malaria in primiparae and the reduced risk of placental infection in multiparae that was found in the recently published paper from Gambia [34] and Malawi [36] was not confirmed by a study conducted in Sudan [33].

One of the main findings of the present study is that severe anaemia is the predominant presenting manifestation of severe *P. falciparum* malaria observed during pregnancy in the Southern part of Saudi Arabia. With the exception of low temperatures, other clinical and biochemical criteria were not different amongst women with different criteria of severe *P. falciparum* malaria. Interestingly however, severe anaemia had been observed as the presenting manifestation of severe *P. falciparum* malaria in pregnant women in central and eastern Sudan [37, 38].

Pro-inflammatory cytokines, especially TNF, IFN-γ and lymphotoxin (LT), have been associated with severe malaria disease and are still crucial for the initial control of parasitaemia in human. The mechanisms are not yet well understood, but secretion of IFN-γ by NK cells, and γδT-cells may have cytotoxic effects on parasite growth as well as activate monocytes and macrophages and enhance non-opsonic phagocytosis of iRBCs. The present study shows that higher levels of TNF and IFN-γ were increasing significantly in UM and SM patients compared to MFC. This observation in SM suggests an imbalanced production of inflammatory cytokines that could contribute to severe malarial anaemia and higher parasite density associated with SM. TNF is an important cytokine in malaria within pregnancy and its levels must be carefully regulated. Low levels are necessary for enhancing phagocytic activity and controlling parasite densities [39, 40], but high levels of TNF can trigger inhibition of endocrine function and initiate extracellular matrix degradation, resulting in poor pregnancy outcomes [41, 42].

High concentrations of TNF are related to the pathogenesis of symptoms associated with malaria infection, such as fever and severe forms of infection, such as cerebral malaria [43, 44]. The biological activity of TNF can be modulated, in part, by its soluble receptors, sTNFR1 and sTNFR2 [45] that are shed from the surface of a number of different cell types by proteolysis. The soluble form of the receptors can stabilize TNF when the cytokine is present in low concentrations in plasma or neutralize TNF by competing for occupation of the receptors on the cell surface when the cytokine is in excess in the local environment [46]. Another suggestion is that the malaria infection also involves elevated production of IgE antibodies [47]. However, IgE-containing immune complexes are pathogenic rather than protective by cross-linking IgE receptors (CD23) on monocytes, leading to local overproduction of TNF, a major pathogenic factor in this disease [47]. T cells are essential for both acquisition and regulation of malaria immunity [47].

The study observed that the IFN-γ cytokine was statistically significantly higher in SM patients compared to UM and MFC. Available studies in both humans and mice have demonstrated the importance of an early IFN-γ response as a crucial determinant in the outcome of the infection and the well-being of the patient [48]– [50]. However, IFN-γ has also been associated with the presence of potent anti-parasitic activity, and persistent high levels of the cytokine can lead to a rapid improvement in fever and reduction in parasitaemia [51, 52]. The present results suggest that the increased levels of IFN-γ seen in infected patients may play a critical role in susceptibility to severe malaria in these groups. In particular, the production of endogenous pyrogens such as TNF, IL-1β and IL-6 following IFN-γ stimulation of monocytes/macrophages may be responsible for fever induction [53] and sustained IL-1β production may be associated with anaemia [54]. The potential roles of pro-inflammatory cytokines in pathology in the setting of maternal malaria are reviewed elsewhere [6].

Population differences in susceptibility or resistance to malaria according to *TNF* SNPs may be a result of diverse evolutionary pressure between ethnicity, as well as different parasite strains and incidence of severe forms of disease. A recent study in sympatric ethnic groups living in Mali suggested that a few *TNF* diplotypes showed an interesting potential influence on severity rather than susceptibility to infection [26]. Previous studies suggested that higher levels of TNF cytokine have been correlated with malaria severity and death [55, 56], and several SNPs in the promoter region of the *TNF* gene have been associated with different outcomes and severity of a malaria infection [57]– [60]. The only recent study that analysed the association between *TNF*-863 C/A polymorphism and susceptibility to malaria infection, suggested that *TNF*-863 CC genotype was statistically significantly associated with malaria infection in sympatric ethnic groups living in Mali [26]. The data presented in this report indicates that the homozygous *TNF*-863 CC genotype was significantly associated with higher levels of TNF cytokine. A previous study in Sweden suggested that individuals carrying *TNF*-863 CC genotype have a significantly higher serum TNF level. However, donors carrying the *TNF*-863 AA have a significantly lower serum TNF-cytokine level [61].

With regards to the *IFN*-*γ*-1616 TT genotype, it is statistically significantly associated with SM patients. Additionally, the *IFN*-*γ*-1616 TT genotype is associated with increased serum concentration of IFN-γ cytokine. A previous study suggested that the *IFN*-*γ* promoter polymorphisms that increase the gene expression may increase IFN-γ levels [62]. The results in this study provide evidence that the *IFN*-*γ*-1616 TT genotype is associated with higher level of IFN-γ among SM patients compared to UM patients and MFC group respectively.

## Conclusions

Taken together, the allele frequency of *TNF*-836 C and *IFN*-*γ*-1616 T genes were statistically significantly higher in those with severe malaria infection compared to uncomplicated malaria patients. Furthermore, *TNF*-836 CC and *IFN*-*γ*-1616 TT genotypes were associated with higher serum concentration of TNF and IFN-γ, respectively, and with susceptibility to severe malaria. This data provides a starting point for functional and genetic analysis of the *TNF* and *IFN*-*γ* genomic region in malaria infection affecting Saudi populations.
